# Institutional Notes and News

**Published:** 1910-02-12

**Authors:** 


### Institutional Notes and News.

**New Lewes Hospital: Opening.**

H.R.H. Princess Henry of Battenberg was present at Lewes on February 2, and opened the new Royal Victoria Hospital. Before the formal opening took place Mr. F. B. Whitfield, J.P. (chairman of directors), made an interesting speech, in which he described the genesis of the scheme for the new institution, the opposition it had met with, and the financial policy by which it was hoped it would pay its way in the future. He pointed out that as no suitable site was available in Lewes itself, one had to be bought outside the town, where the dispensary was still kept open, and this at once raised the question of expense. Building, road-making, the raising of walls, and so on, had cost about £6,170, of which £6,142 already had been subscribed. For furnishing £1,000 was needed, towards which £150 was now in hand. In order to maintain the hospital for more than the one year to which its opponents had limited its life, annual subscriptions were wanted, and these he appealed for as a basis of income. Some people had said the new hospital was not wanted, but he was sure the majority of Lewes' citizens did not agree with that. Everyone knew what their present hospital was. Mr. Whitfield concluded by saying that without Lady Gage they would never have started. He then presented a silver key to Her Royal Highness, who, after formally opening the building, signed her name in the visitors' book.

**Dr. Brown, of Preston.**

Preston Infirmary has a good friend in Dr. Brown, who helped to bring the Institution thoroughly up-to-date in every respect. He has defrayed the expense of building a new operating theatre costing about £2,700. Some time ago he presented the Infirmary with a beautiful organ, built by Messrs. Ainscough, of Preston. He followed the gift up with a piano and pianola, and, not forgetting the staff, he fitted up a billiard-room for the medical staff, and provided a bagatelle board and other games for the nurses' recreation-room. He subsequently caused to be erected at his own expense a conservatory for convalescent patients. In 1903 he defrayed the cost of a complete installation of *x*-ray apparatus. To this apparatus he has added a Finsen light. As a memorial to his sister he erected a second conservatory to be used as a smoke-room, and the one previously given by him became a day-room for convalescent female patients. He also established two revolving summer houses for the open-air treatment of children, and a conservatory to be occupied by them on wet days. Dr. Brown's latest gift is a building which is to be used for the temporary isolation of infirmary in-patients. These observation wards are being erected according to the plans of Mr. F. E. Dixon, and form a one-story, detached building connected by a covered way open at the sides with the existing isolation cottage facing Deepdale Road. They are three in number, two 10 feet square and one 12 feet by 10 feet, and are fitted with vestibules. A verandah runs along the front of the building, and a small lavatory is provided. The floors are of concrete, laid to a fall for convenience in flushing out, and the walls and ceiling are Portland cement, finished in Parian. The angles of the walls, floors, and ceilings are rounded to prevent lodgment of dust, and all mouldings in woodwork are avoided. The buildings are being built of Huncoate brick, with Longridge stone dressings. Fireplaces are provided, and also radiators, which latter are intended to be constantly in use, so that the wards will always be aired.

**Chester General Infirmary.**

The Governors of Chester General Infirmary held their annual meeting last week. They were able to say that the invested funds have been increased by £5,244, owing to legacies received. The balance sheet, therefore, read as follows: To the credit side, which included gifts to the convalescent home, £13,000 odd; while the sum debited to ordinary expenditure was £6,211. The report went on to remark that Sir Henry Burdett, "in the course of a voluntary inspection," had given as his opinion that as it was 150 years since the hospital was built every effort should be made to provide a new one. The Board felt it its duty to state that £60,000 would be necessary for this purpose. The daily average number of beds occupied is 97, the average cost per bed occupied, £56 18s. 4d.; and the number of patients treated, 9,295. The Mayor (Mr. Alderman Hewitt), who presided, said that medical men who had read Sir Henry's comments in the visitors' book, remarked that many of these, unfortunately, were true. Mr. Hewitt then warned the citizens to be prepared for something drastic, a sign no doubt that The Hospital's report is being awaited with interest.

**Glasgow Eye Infirmary.**

Sir James King, Bart., LL.D., presided over the eighty-ninth annual meeting in the Merchants' House on January 26. Over 24,000 cases have been treated during the year, and the daily average number of patients was 82.48, as compared with 78.6 in the previous year. Owing to increasing work performed by the institution the number of nurses has been increased, and an honorary medical electrician and two honorary anæsthetists have been appointed. Last year's income was £4,303, or £216 less than in 1908. This deficit was attributed to the prevailing unemployment in the city, the contributions from public works alone being less by £159 than previously. Ordinary expenditure totalled £5,482, £74 less than in 1908, but they had to draw upon the extraordinary revenue for £1,179. The yearly report records two serious losses by death during the year.

**Some Losses.**

The sudden and unexpected death of Mr. Bergius deprived the infirmary of an ideal chairman of the House Committee, while another serious loss was sustained by the death of the senior surgeon, Dr. Meighan, who had been a member of the staff for 36 years, and who was equally successful as an operator and as an instructor. The Chairman referred to the retirement of Mr. William George Black after filling the office of secretary for 25 years. Mr. Black's efficiency and zeal for the prosperity of the institution were only equalled by his characteristic courtesy, and now that he was relieved from the duties of his profession it was to be trusted that he would find ample opportunity for the public services he was willing to render. Mr. R. G. Paterson, hon. treasurer, submitted the financial statement, and the reports, on being seconded by Mr. J. J. Spencer, were unanimously adopted. Votes of thanks were awarded to the medical officers and staff, the secretary and acting treasurer, the honorary treasurer, retiring directors, lady visitors, the Kyrle Society, the chairman of the House Committee (Mr. Spencer), the operatives in Glasgow and neighbourhood, and the parishes, corporations, and societies who had contributed to the funds of the infirmary, and to all who had assisted in brightening the lot of the patients during their residence in the institution.

**Bradford Infirmary.**

The Board of Governors of the Bradford Royal Infirmary last week inspected the Field House estate at Daisy Hill, which has been recommended by the Selection Committee as the site for the new Infirmary. The estate, which is about 23 acres in extent and includes a large residence, was occupied for many years by the late Mr. Isaac Smith, and negotiations have been in progress with his executors. After the inspection the Board met at the Royal Infirmary and after discussing the proposal at considerable length, decided to adopt the recommendation and to purchase the site. The Royal Infirmary is therefore likely to be removed entirely to Daisy Hill in the course of the next few years, and that will no doubt bring into the market the site at present occupied at the junction of Lumb Lane with Westgate. The purchase price of the estate is not to be disclosed, but it is understood, says a local paper, that advantageous terms have been arranged.

**Southwold Cottage Hospital.**

The annual meeting of the subscribers to the Southwold Cottage Hospital, near Ipswich, was as usual crowded, and the lengthy minutes, which included remarks of speakers at the last meeting, caused so much contention that only that part of them which referred to business was passed. Lord Stradbroke, who presided, then referred to the hospital's by-laws and constitution, which provoked plenty of disagreement. The point at issue appeared to be whether a patient who desired his own doctor could have him admitted to the hospital. The by-laws said "by arrangement with the hon. medical staff," and this rule had been laid down owing to the relations between certain medical men. The by-laws were eventually passed, but Mrs. Foster, the hon. secretary, said that if she accepted re-election that must not imply that she approved of the rules. Ultimately she agreed to remain in office for three months. The thirteenth annual report stated that for the first time the number of patients treated had reached one hundred.

**Bristol Eye Hospital.**

The centenary of the Bristol Eye Hospital is to be celebrated this year by a fête to be held in the Zoological Gardens in June. It is hoped that this will result in a satisfactory sum being handed over to the treasurer, for the ninety-ninth annual report, which was adopted at the annual meeting on February 2, showed a loss of £1,100 on the year's work. The chief factor in the income of the hospital is £500 derived from invested legacies, but the subscription list must be raised to £1,000 if the hospital is really to pay its way. The in-patients numbered 441 for the year, the out-patients 6,828, and the number of attendances totalled 18,879. Though the city of Bristol supplies, of course, the chief share of patients, some fourth or sixth part comes from the surrounding districts. Taunton and Wellington, Gloucester and Cheltenham, Bath and Chippenham, all send a share, and even South Wales, the Lord Mayor pointed out, can generally be counted on! This being so the hospital authorities think it is only fair that the list of subscribers should cover a wider area than the city itself. The Lord Mayor suggested at the meeting that all the religious denominations of the locality should be reminded that Bristol has its own Hospital Sunday, and that every church or chapel should provide at least one collection on that day for his fund. The thanks of the institution were recorded for the use of the pathological laboratory of the Bristol Royal Infirmary. The Eye Hospital has grown so much in recent years, to meet the increasing and dispersed demands upon it, that in the words of the report, "it would seem just that the expenses of a patient should fall chiefly on the district whence he comes."

**Cottage Hospitals and Out-patients.**

The Right Hon. Sir J. H. Kennaway, Bart., presided over the 16th annual meeting of the subscribers to the Ottery St. Mary District Cottage Hospital, Devon. Owing to the resignation of the matron, after nine years' good work, the committee have secured a highly trained nurse and two probationers, thus avoiding the expense of an occasional night nurse. An enterprising and interesting experiment has also been made by which an out-patient department has been started; it is hoped the poor, and especially the poor children, will thus be still better medically provided for. The only regrettable fact mentioned in the report was a deficit of £47.

